# Interaction between autophagy and senescence is required for dihydroartemisinin to alleviate liver fibrosis

**DOI:** 10.1038/cddis.2017.255

**Published:** 2017-06-15

**Authors:** Zili Zhang, Zhen Yao, Shifeng Zhao, Jiangjuan Shao, Anping Chen, Feng Zhang, Shizhong Zheng

**Affiliations:** 1 Department of Pharmacology, School of Pharmacy, Nanjing University of Chinese Medicine, Nanjing, China; 2 Department of Pathology, School of Medicine, Saint Louis University, St Louis, MO, USA; 3 Jiangsu Key Laboratory for Pharmacology and Safety Evaluation of Chinese Materia Medica, Nanjing University of Chinese Medicine, Nanjing, China; 4 Jiangsu Key Laboratory of Therapeutic Material of Chinese Medicine, Nanjing University of Chinese Medicine, Nanjing, China

## Abstract

Autophagy and cellular senescence are stress responses essential for homeostasis. Therefore, they may represent new pharmacologic targets for drug development to treat diseases. In this study, we sought to evaluate the effect of dihydroartemisinin (DHA) on senescence of activated hepatic stellate cells (HSCs), and to further elucidate the underlying mechanisms. We found that DHA treatment induced the accumulation of senescent activated HSCs in rat fibrotic liver, and promoted the expression of senescence markers p53, p16, p21 and Hmga1 in cell model. Importantly, our study identified the transcription factor GATA6 as an upstream molecule in the facilitation of DHA-induced HSC senescence. GATA6 accumulation promoted DHA-induced p53 and p16 upregulation, and contributed to HSC senescence. By contrast, siRNA-mediated knockdown of GATA6 dramatically abolished DHA-induced upregulation of p53 and p16, and in turn inhibited HSC senescence. Interestingly, DHA also appeared to increase autophagosome generation and autophagic flux in activated HSCs, which was underlying mechanism for DHA-induced GATA6 accumulation. Autophagy depletion impaired GATA6 accumulation, while autophagy induction showed a synergistic effect with DHA. Attractively, p62 was found to act as a negative regulator of GATA6 accumulation. Treatment of cultured HSCs with various autophagy inhibitors, led to an inhibition of DHA-induced p62 degradation, and in turn, prevented DHA-induced GATA6 accumulation and HSC senescence. Overall, these results provide novel implications to reveal the molecular mechanism of DHA-induced senescence, by which points to the possibility of using DHA based proautophagic drugs for the treatment of liver fibrosis.

Liver fibrosis is a reversible wound-healing response following liver injury, and its end-stage cirrhosis is responsible for high morbidity and mortality worldwide.^1, 2, 3^ Liver transplantation is the only treatment available for patients with advanced stages of liver fibrosis.^4, 5, 6^ Therefore, new therapeutic agents and strategies are needed for the management of this disease.^7, 8^ Dihydroartemisinin (DHA), a natural and safe anti-malarial agent, exhibits an ample array of pharmacological activities such as anti-tumor,^9^ anti-bacterial^10^ and anti-schistosomiasis properties.^11^ We previously reported that DHA treatment improved the inflammatory microenvironment of liver fibrosis *in vivo*,^12^ and inhibited activation and contraction of hepatic stellate cells (HSCs) *in vitro.*^13, 14, 15, 16^ In the current study, we aimed to evaluate the effect of DHA on HSC senescence and to further elucidate the underlying mechanisms.

Cellular senescence is a terminal arrest of proliferation triggered by various cellular stresses including dysfunctional telomeres,^17^ DNA damage^18^ and oncogenic mutations.^19^ Cellular senescence not only prevents the proliferation of damaged cells, thereby preventing tumorigenesis, but also affects the microenvironment through the secretion of pro-inflammatory cytokines, chemokines, and proteases, a feature termed the senescence-associated secretory phenotype (SASP).^20^ The mechanisms underlying induction and maintenance of cell senescence remain entirely elusive.^21, 22, 23^ Previous studies^21, 22^ have reported that p53 can lead to cell cycle arrest, DNA repair and apoptosis predominantly when it becomes transcriptionally active in response to DNA damage, oncogene activation and hypoxia. Retinoblastoma 1 (pRb) inactivation mediated by p16 is also known to ensure durable cell cycle arrest, but is unlikely to be regulated by a canonical DNA damage response.^23^ Attractively, it is interesting to explore the mechanism underlying the induction and maintenance of cell senescence in liver fibrosis.

Interestingly, several lines of evidence indicate a genetic relationship between autophagy and senescence.^24, 25^ However, whether autophagy acts positively or negatively on senescence is still subject to debate.^25^ Through a specialized compartment known as the TOR-autophagy spatial coupling compartment (TASCC), autophagy generates a high flux of recycled amino acids, which are subsequently used by mTORC1 for supporting the massive synthesis of the SASP factors and facilitating senescence.^25^ In contrast, increased levels of reactive oxygen species upon autophagy inhibition partially contribute to cellular senescence.^25^ We previously reported that DHA treatment stimulated autophagy activation via a ROS-JNK1/2-dependent mechanism in liver fibrosis.^12^ Attractively, whether autophagy activation contributes to DHA-induced HSC senescence is worth to further study.

In the present study, we evaluated the effect of DHA on HSC senescence, and to further elucidate the underlying mechanisms. We found that DHA could induce senescence of activated HSCs to alleviate liver fibrosis via autophagy-dependent GATA6 accumulation. The results of the present study provide important information concerning the molecular mechanisms that underlie the antifibrotic activities of DHA, which is essential for investigating its potential for clinical application.

## Results

### DHA induces the accumulation of senescent activated HSCs in rat fibrotic liver

**Figure 1:**
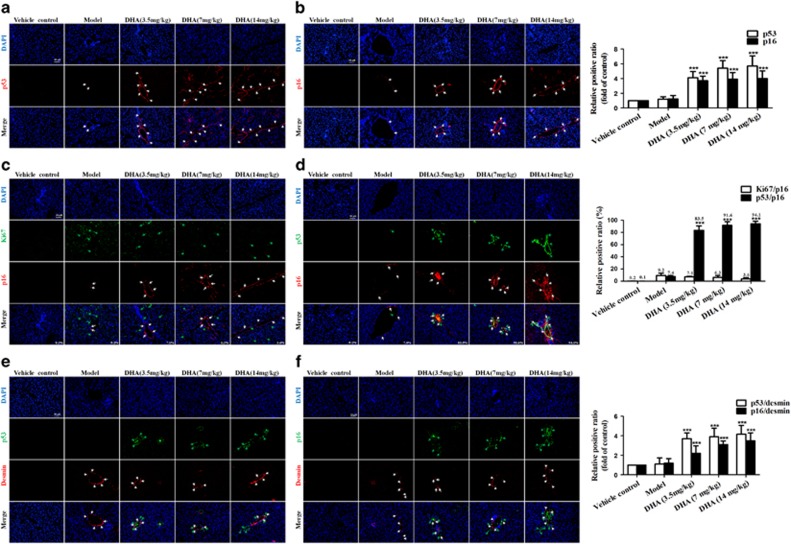
DHA induces the accumulation of senescent activated HSCs in rat fibrotic liver. Rats were grouped as follows: group 1, vehicle control (no CCl_4_, no treatment); group 2, model group (with CCl_4_, no treatment); group 3, DHA (3.5 mg/kg) and CCl_4_-treated group; group 4, DHA (7 mg/kg) and CCl_4_-treated group; group 5, DHA (14mg/kg) and CCl_4_-treated group. (**a** and **b**) Liver sections were stained with immunofluorescence by using antibodies against p53 and p16. White arrows indicated p53 and p16-positive cells. (**c**) Liver sections were co-stained with p16 and Ki67. White arrows indicated p16-positive cells, while green arrows indicated Ki67-positive cells. (**d**) Liver sections were co-stained with p16 and p53. White arrows indicated p16-positive cells, while green arrows indicated p53-positive cells. (**e**) Liver sections were co-stained with desmin and p53. White arrows indicated desmin positive cells, while green arrows indicated p53-positive cells. (**f**) Liver sections were co-stained with desmin and p16. White arrows indicated desmin positive cells, while green arrows indicated p16-positive cells. For the statistics of each panel in this figure, data are expressed as mean±S.D. (*n*=6). Scale bars are 50 *μ*m. For the statistics of each panel in this figure, data are expressed as mean±S.D. (*n*=6); *P<0.05 *versus* control, **P<0.01 *versus* control, ***P<0.001 *versus* control

Our previous data^12, 13, 14, 15, 16^ and the present results (Supplementary Figures 1A–C) have sufficiently demonstrated that DHA protected the liver against CCl_4_-induced injury and suppressed hepatic fibrogenesis in the rat model. To investigate the mechanisms underlying the protective effects of DHA, we proposed that DHA might induce senescence of activated HSCs to limit liver fibrosis. To identify senescent cells *in situ*, we stained liver sections from DHA and vehicle-treated rat for a panel of senescence-associated markers, including SA-*β*-gal, p53 and p21. Results from immunofluorescence staining showed that cells staining positive for each senescence-associated markers accumulated in fibrotic livers, and were invariably located along the fibrotic scar by treatment with DHA in a dose-dependent manner (Figures 1a and b; Supplementary Figure 1D). Interestingly, we also found that these cells typically expressed multiple senescence markers and were not proliferating. As shown in Figures 1c and d, of the p16-positive cells identified in DHA-treated livers, more than 80% were positive for p53 staining, whereas less than 9% co-expressed the proliferation-association marker Ki67. Although hepatocytes represent the most abundant cell type in the liver, the location of senescent cells along the fibrotic scar in rat livers raised the possibility that these cells were derived from activated HSCs, which initially proliferate following liver damage and are responsible for much of the extracellular matrix production in fibrosis.^4, 5, 6^ In order to verify this hypothesis, the cells in DHA- and vehicle-treated liver sections were not only stained positive for the senescence-associated markers p53 and p16, but also were positive for the HSC marker desmin. As expected, cells expressing the senescence markers p53 and p21 co-localized with those expressing desmin (Figures 1e and f). Overall, these results indicate that DHA induces the accumulation of senescent activated HSCs in rat fibrotic liver.

### DHA promotes activated HSC senescence *in vitro*

**Figure 2:**
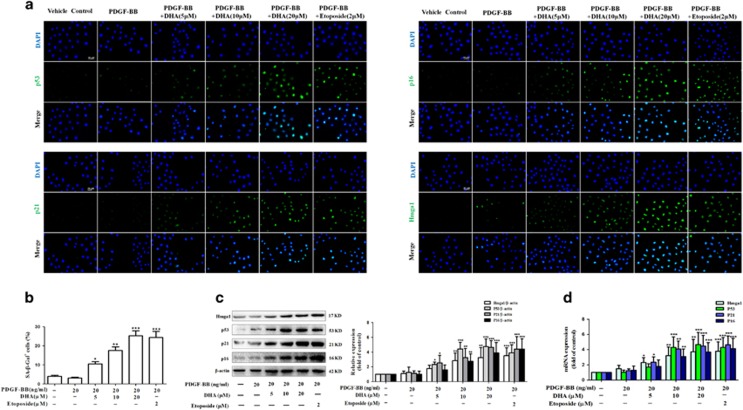
DHA promotes activated HSC senescence *in vitro*. Primary HSCs were exposed for 6 h to 20 ng/ml PDGF-BB followed by vehicle, Etoposide or DHA treatment at indicated concentrations for 24 h. (**a**) Immunofluorescence using antibody against p53, p16, p21 and Hmga1. DAPI was used to stain the nucleus. Scale bars are 50 *μ*m. (**b**) Senescence *β*-galactosidase staining was used to detect senescence. (**c** and **d**) Western blot and Real-time PCR analysis were used to determine the expression of Hmga1, p53, p21 and p16. Representative blots were from three independent experiments. For the statistics of each panel in this figure, data are expressed as mean±S.D. (*n*=3); *P<0.05 *versus* control, **P<0.01 *versus* control, ***P<0.001 *versus* control

Previous studies have confirmed that HSC activation *in vivo*, as a result of different liver injuries, could be mimicked *in vitro* by plating freshly isolated HSCs exposed to platelet derived growth factor-BB (PDGF-BB) on plastic tissue culture dishes.^12, 13, 14, 15, 16, 26^ Therefore, the freshly quiescent HSCs were isolated from Sprague-Dawley rats as described,^27^ and then were treated with 5, 10 and 20 ng/ml PDGF-BB. In agreement with previous findings,^12, 13, 14, 15, 16^ HSC activation markers like *α*-SMA (acta-2), Fibronectin, Procollagen 1*α*1 (procol1*α*1), TNF-*α*, and TGF-*β* were significantly upregulated showing that HSCs undergo an activation process *in vitro* as well (Supplementary Figures 2A–C). Subsequently, we used cultured HSCs to test whether DHA treatment could promote activated HSC senescence *in vitro*. Immunofluorescent assay showed that DHA and Etoposide (as a positive control)^28^ treatment significantly increased the expression of senescence markers p53, p16, p21, and Hmga1 in cell model (Figure 2a). Besides, we found that DHA treatment increased the number of SA-*β*-Gal-positive HSCs (Figure 2b). Western blot and Real-time PCR analysis of senescence-associated proteins also consistently showed that DHA treatment upregulated the expression of p53, p16, p21 and Hmga1 in activated HSCs (Figures 2c and d).

Additional experiments were performed to verify the role of telomerase activity in DHA-induced HSC senescence. We found that the telomerase activity was decreased in DHA-treated HSCs (Supplementary Figure 2D). A well-known feature of cellular senescence is cell cycle arrest, which largely accounts for the growth inhibition in senescent cells.^20^ Next, we examined the cell cycle distribution by a flow cytometer. As shown in Supplementary Figures 2E and F, HSCs treated with DHA or Etoposide showed significantly higher proportions of G2/M cells and lower proportions of S cells compared with untreated HSCs. Cell cycle is influenced by multiple cyclins and cyclin-dependent kinases (CDKs).^29^ Real-time PCR analyses indicated that DHA treatment downregulated the expression of cyclin D1, cyclin E1 and CDK4 in activated HSCs (Supplementary Figure 2G). Taken together, these results show that DHA promotes activated HSC senescence *in vitro*.

### The accumulation of GATA6 is required for DHA to induce activated HSC senescence *in vitro*

**Figure 3:**
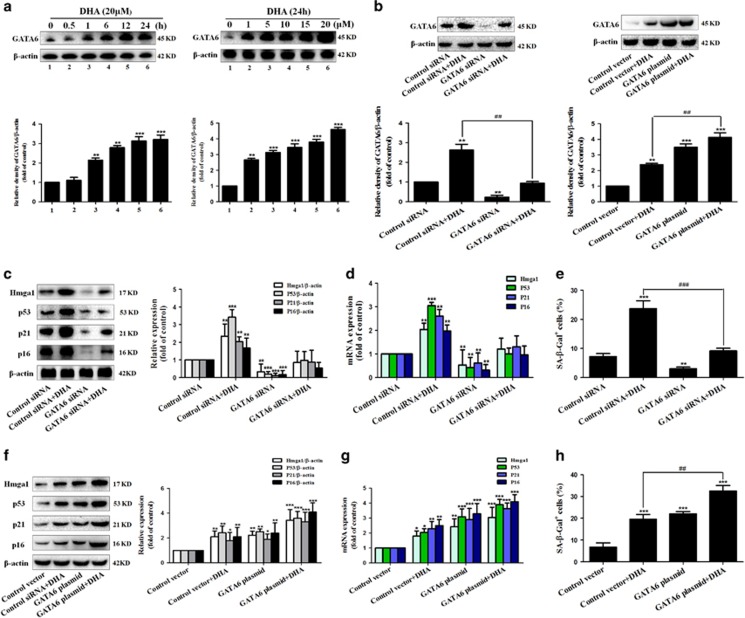
The accumulation of GATA6 is required for DHA to induce HSC senescence *in vitro*. (**a**) Activated HSCs were treated with DHA at various concentrations for 24 h or DHA at 20 *μ*M for various hours. Then, western blot was used to determine the expression of GATA6. Activated HSCs were stably transfected with GATA6 siRNA or GATA6 plasmid, and then were treated with 20 *μ*M DHA for 24 h. (**b**) Western blot was used to determine the expression of GATA6. (**c**, **f**) Western blot was used to determine the expression of Hmga1, p53, p21 and p16. (**d**, **g**) Real-time PCR was used to show the mRNA expression of Hmga1, p53, p21 and p16. (**e**, **h**) *β*-galactosidase staining was used to detect senescence. For the statistics of each panel in this figure, data are expressed as mean±S.D. (*n*=3); *P<0.05 *versus* control, **P<0.01 *versus* control, ***P<0.001 *versus* control. ^#^*P*<0.05 *versus* DHA treatment, ^##^*P*<0.01 *versus* DHA treatment, ^###^*P*<0.001 *versus* DHA treatment

Cellular senescence is a terminal stress-activated program mainly controlled by the p53 and p16^INK4a^ tumor suppressor proteins.^22, 23^ However, in contrast to the downstream functionality of p53 and p16, its upstream control is a relatively unexplored area.^30^ In the current study, we hypothesized that GATA6 could have a pivotal role in DHA-induced upregulation of p53 and p16 in activated HSCs. To test this hypothesis, the status of this GATA6 protein was evaluated following the DHA treatment. As shown in Figure 3a, DHA treatment obviously increased the level of GATA6 in a time- and dose-dependent manner. In order to further detect the role of GATA6 accumulation in the DHA-induced senescence, activated HSCs were pre-treated with GATA6 siRNA or GATA6 plasmid, followed by DHA treatment (Figure 3b). As expected, the results from SA-*β*-Gal staining showed that pretreatment with GATA6 siRNA significantly abrogated DHA-induced increase of SA-*β*-Gal-positive HSCs, whereas GATA6 plasmid showed a synergistic effect with DHA (Figures 3e and h). Besides, in order to investigate the effect of GATA6 accumulation on DHA-induced p53 and p16 upregulation, the expression of p53, p16, and their downstream effectors were detected by western blot and Real-time PCR analysis. The results revealed that GATA6 plasmid, mimicking DHA, promoted the expression of p53, p21, Hmga1 and p16, while GATA6 siRNA dramatically suppressed the ability of DHA and GATA6 plasmid in inducing cellular senescence (Figures 3c, d, f and g). Furthermore, immunofluorescent assay also indicated that DHA as well as GATA6 plasmid significantly increased the abundance of proteins involved in senescence (Supplementary Figures 3B and D). However, the pretreatment of cells with GATA6 siRNA dramatically eliminated the promoting effects of DHA on the expression of p53 and p16 in activated HSCs (Supplementary Figures 3A and C). Attractively, accumulating evidence suggests that mitogen activated protein kinases (MAPKs) have important roles in the activation of p53 and p16.^31, 32, 33^ Thus, it was assumed that GATA6 accumulation contributed to DHA-induced upregulation of p53 and p16 via a MAPK-dependent mechanism. To test this assumption, the phosphorylation status of these MAPK proteins was evaluated following the GATA6 siRNA or GATA6 plasmid treatment. The results showed that GATA6 plasmid obviously increased the level of phosphorylated JNK1/2, but did not significantly affect the level of phospho-ERK1/2 and phospho-p38 (Supplementary Figure 3E), suggesting the involvement of JNK1/2 in GATA6-induced upregulation of p53 and p16. In order to further determine the association between GATA6 accumulation and p53 or p16 upregulation, selective JNK1/2 inhibitor (SP600125) was used to inhibit the activity of JNK1/2. Interestingly, we found that pretreatment with SP600125 abolished GATA6 plasmid or DHA-induced p53 and p16 upregulation (Supplementary Figures 3F and G), demonstrating that JNK1/2 could mediate p53 and p16 upregulation induced by GATA6 accumulation. Collectively, these data demonstrate that the accumulation of GATA6 is required for DHA to induce HSC senescence *in vitro*.

### DHA induces HSC senescence via a GATA6-dependent mechanism *in vivo*

**Figure 4:**
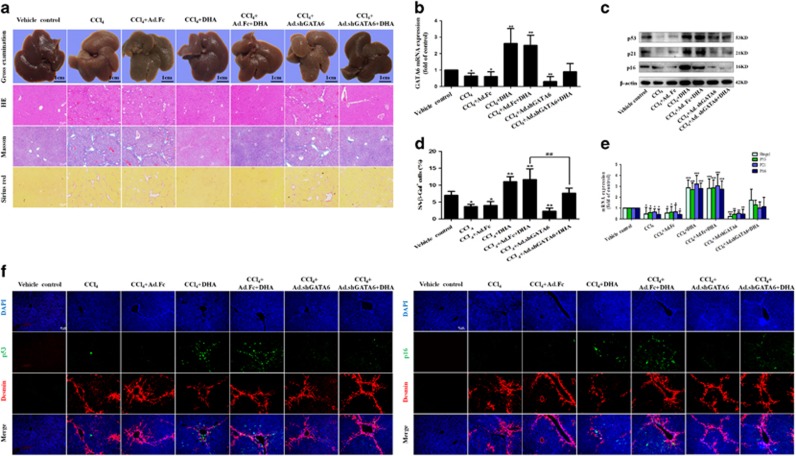
DHA induces HSC senescence via a GATA6-dependent mechanism *in vivo*. Mice were randomly separated into seven groups: group 1, vehicle control (no CCl_4_, no treatment); group 2, model group (with CCl_4_, no treatment); group 3, Ad.Fc (a control adenovirus encoding IgG2 *α* Fc fragment) and CCl_4_-treated group; group 4, DHA (20 mg/kg) and CCl_4_-treated group; group 5, Ad.Fc, DHA and CCl_4_-treated group; group 6, Ad.shGATA6 (adenovirus encoding mouse GATA6 shRNA for inhibiting GATA6 expression) and CCl_4_-treated group; group 7, Ad.shGATA6, DHA and CCl_4_-treated group. (**a**) The pathological changes of the liver were observed by Gross examination, Scale bars are 1cm. Liver sections were stained with hematoxylin and eosin, Masson reagents and Sirius red. Representative photographs are shown. (**b**,**c**,**e**) Primary HSCs were isolated and subsequently were used to determine the expression of GATA6, p53, p21 and p16. (**d**) Liver sections were stained with *β*-galactosidase. (**f**) Liver sections were co-stained with desmin and p53 or p16. For the statistics of each panel in this figure, data are expressed as mean±S.D. (*n*=6); *P<0.05 *versus* control, **P<0.01 *versus* control, ***P< 0.001 *versus* control. ^##^*P*<0.01 *versus* CCl_4_+DHA+Ad.Fc treatment

We further examined whether the disruption of GATA6 accumulation could affect DHA-induced upregulation of p53 and p16 *in vivo*. Seventy mice were randomly divided into seven groups of ten animals each with comparable mean bodyweight. Mice of seven groups were administrated with vehicle control, CCl_4_, CCl_4_+Ad.Fc, CCl_4_ +DHA, CCl_4_+Ad.Fc+DHA, CCl_4_+Ad.shGATA6 or CCl_4_+Ad.shGATA6+DHA, respectively, throughout the 8-week period of CCl_4_ treatment. First and foremost we evaluated the effect of interrupting GATA6 on liver injury *in vivo*. Gross examination showed that morphological changes pathologically occurred in the mouse liver exposed to CCl_4_ compared with the normal liver, but DHA treatment improved the pathological changes in livers (Figure 4a). Interestingly, the improvement of DHA on liver injury was remarkably abrogated by Ad.shGATA6 (Figure 4a). Besides, liver fibrosis was also demonstrated by histological analyses. Hematoxylin and eosin (H&E), Masson and picro-Sirius red staining showed that intraperitoneal injection of DHA daily for 4 weeks significantly improved histopathological feature of liver fibrosis characterized by decreased collagen deposition, whereas livers derived from mice treated with DHA plus Ad.shGATA6 exhibited more severe liver fibrosis compared with the mice treated with DHA alone (Figure 4a). Next, primary HSCs were isolated for detection of cell senescence markers. The Real-time PCR analysis showed that Ad.shGATA6 significantly reduced the GATA6 level of activated HSCs (Figure 4b). Then, western blot and Real-time PCR analysis demonstrated that interference of GATA6 significantly inhibited the expression of p53, p21 and p16, suggesting that the effect of DHA was at least partially reversed (Figures 4c and e). Besides, Ad.shGATA6 treatment not only decreased the number of SA-*β*-Gal-positive cells, but also markedly eliminated the regulatory effects of DHA on cell senescence (Figure 4d). More importantly, liver tissues were co-stained with the senescence markers p53 or p16 and HSC activation marker desmin. Results from immunofluorescence staining showed that DHA induced the accumulation of senescent activated HSCs in fibrotic liver, whereas Ad.shGATA6 treatment impaired the induction of DHA on activated HSC senescence (Figure 4f). Altogether, these data suggest that GATA6 accumulation is involved in DHA-induced HSC senescence *in vivo*.

### The activation of autophagy is associated with DHA-induced GATA6 accumulation and HSC senescence

**Figure 5:**
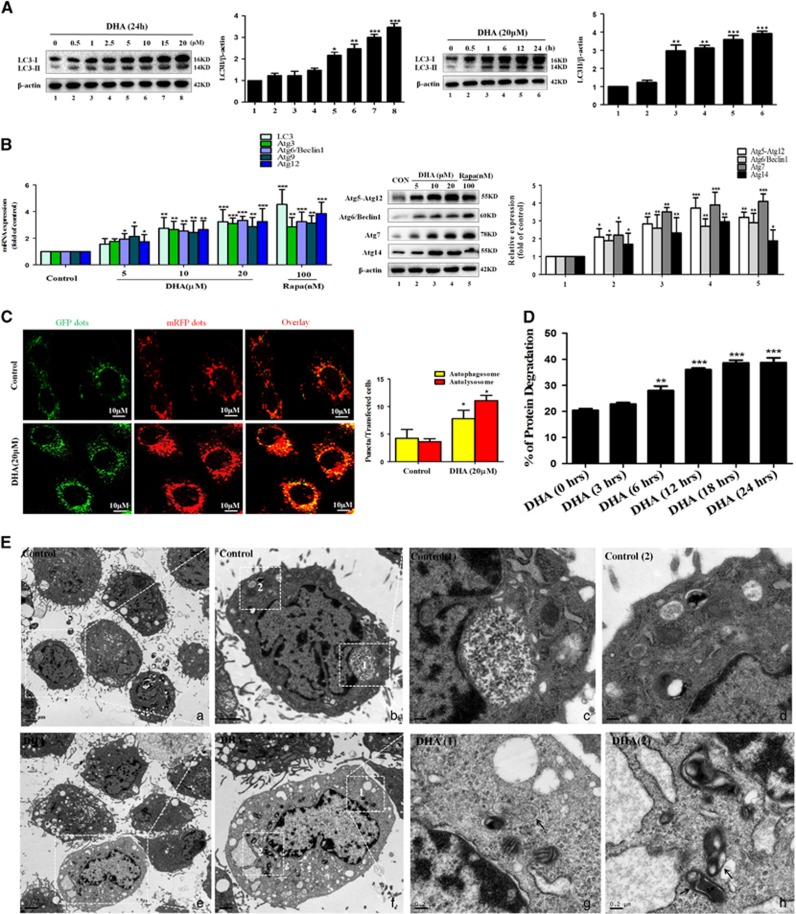
The activation of autophagy is associated with DHA-induced GATA6 accumulation and HSC senescence. Activated HSCs were treated with DHA at various concentrations for 24 h or DHA at 20 *μ*M for various hours. (**A**) Western blot and densitometric analysis were used to show the response of autophagic regulators in activated HSCs. (**B**) Real-time PCR and western blot data were used to show levels of different autophagy related genes in activated HSCs treated with DHA (5, 10 and 20 *μ*M) or Rapa (100 nM) for 24 h compared with *versus* vehicles treated cells (Control). (**C**) Activated HSCs were transfected with a plasmid containing LC3-II tagged at the N terminus with green (GFP) and red fluorescent protein (RFP). This probe allows distinction of early autophagosomes (overlapping GFP+RFP puncta generating yellow puncta on overlay) and late autolysosomes (RFP puncta), as GFP fluorescence is quenched in the acidic autolysosomes. Images were examined under a × 20 lens on an Olympus fluorescence microscope using standard filter sets for GFP and RFP. (**D**) Primary HSCs were treated with DMSO or 20 *μ*M DHA following 20 ng/ml PDGF-BB treatment for indicated hours. Longevity protein degradation was detected by LC–MS/MS. (**E**) Transmission electron microscope (TEM) analysis was used to show increased autophagosomes and autolysosomes in HSCs treated with 20 nM PDGF-BB and 20 *μ*M DHA for 24 h compared with *versus* vehicles treated cells (Control); For the statistics of each panel in this figure, data are expressed as mean±S.D. (*n*=3); *P<0.05 *versus* control, **P<0.01 *versus* control, ***P<0.001 *versus* control

Protein accumulation is controlled by two major pathways in eukaryotic cells: the ubiquitin-proteasome^34^ and autophagy-lysosome pathways.^35^ Interestingly, Kang *et al.*^36^ showed that inhibition of the proteasome by MG-132, a proteasome inhibitor, had no effect on GATA4 abundance, whereas GATA4 protein was stabilized in cells treated with distinct lysosomal inhibitors known to block autophagy. In the present study, we assumed that autophagy-lysosome pathways could have a pivotal role in DHA-induced GATA6 accumulation. To evaluate this assumption, activated HSCs were treated with various concentrations of DHA for 24 h or with 20 *μ*M of DHA for different hours. Results from western blot analysis showed that DHA induced the generation of autophagosome in a dose*-* and time-dependent manner (Figure 5a). Besides, seven important autophagy related genes were detected by western blot and Real-time PCR analysis in DHA- or vehicle-treated cells. The results revealed that DHA treatment increased the level of many indicators of the autophagosome (Figure 5b). Furthermore, immunofluorescence of Atg6/Beclin1 and endogenous LC3-II also proved the facilitating roles of DHA on autophagosome (Supplementary Figures 4C and D). Numerous studies have shown a crucial role for mTOR signaling pathway in autophagosome generation.^37, 38^ Therefore, we evaluated whether DHA treatment affect the expression of p-ULK1, ULK1, p-mTOR and mTOR. Western blot analysis revealed that DHA-induced autophagosome generation was associated with an increase in p-ULK1 activity and a decrease in p-mTOR activity (Supplementary Figure 4A).

Next, we further assessed the effect of DHA on autophagic flux in activated HSCs. Firstly, tandem fluorescence RFP-GFP-LC3 (tf-LC3) staining was used to demonstrate the autophagic flux. It has been documented that, in autophagosomes, the combination of both RFP and GFP in the triple fusion yields yellow fluorescence, whereas autolysosomal delivery results in red.^39^ As expected, we observed that both autophagosome and autolysosome formation were increased in DHA-treated HSCs (Figure 5c). Secondly, the long-lived protein degradation was detected to indicate autophagic flux because it was substrate for autophagy, and the rate was a key functional index of autophagic flux.^39^ The longevity protein degradation rate reflected that DHA time-dependently increased autophagic flux (Figure 5d). Thirdly, we observed an increase in LC3-II level in cells which cultured with DHA for 24 h followed by chloroquine (CQ) treatment compared with cells which were treated with DHA alone, suggesting that autophagic flux is increased in DHA-treated HSCs (Supplementary Figure 4B). Lastly, the transmission electron microscopy (TEM) was used to observe autophagy.^39^ As expected, we observed the presence of a high level of autophagosomes or lysosomes in DHA-treated HSCs. In contrast, it was difficult to observe autophagosomes or lysosomes in control HSCs (Figure 5e). Overall, these results support that DHA increases the autophagosome generation and autophagic flux in activated HSCs.

### Disruption of autophagy impairs DHA-induced GATA6 accumulation and HSC senescence *in vitro*

**Figure 6:**
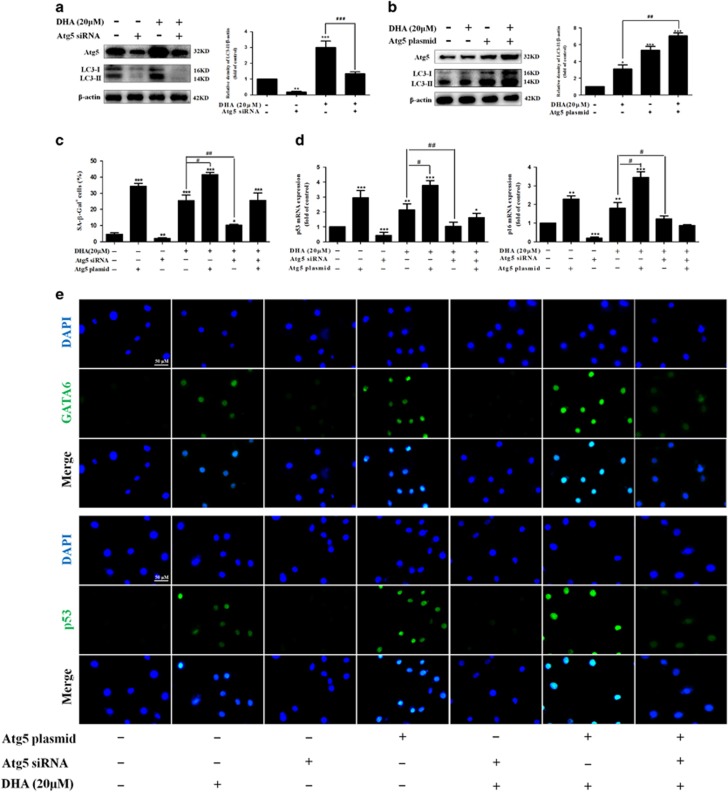
Disruption of autophagy impairs DHA-induced GATA6 accumulation and HSC senescence *in vitro*. Activated HSCs were stably transfected with Atg5 siRNA or Atg5 plasmid, and then were treated with the indicated concentration of DHA for 24 h. (**a**, **b**) The transfection efficiency was confirmed by western blot analysis. (**c**) *β*-galactosidase staining analysis was used to detect HSC senescence. (**d**) Real-time PCR analyses were used to determine the expression of p53 and p16. (**e**) GATA6 and p53 immunostaining were used to determine the expression of GATA6 and p53. For the statistics of each panel in this figure, data are expressed as mean±S.D. (*n*=3); *P<0.05 *versus* control, **P<0.01 *versus* control, ***P<0.001 *versus* control. ^#^*P*<0.05 *versus* DHA treatment, ^##^*P*<0.01 *versus* DHA treatment, ^###^*P*<0.001 *versus* DHA treatment

To determine whether the activation of autophagy by DHA is directly involved in the GATA6 accumulation and HSC senescence *in vitro*, we used Atg5 siRNA to block the autophagosome formation and employed Atg5 plasmid to induce autophagy (Figures 6a and b). Then, SA-*β*-Gal staining was performed to measure its effects on cell senescence. As shown in Figure 6c, Atg5 plasmid, mimicking DHA, increased the number of SA-*β*-Gal-positive HSCs. Conversely, siRNA-mediated knockdown of Atg5 markedly suppressed the ability of DHA and Atg5 plasmid in the induction of cell senescence. Furthermore, Real-time PCR analysis indicated that the pretreatment of cells with Atg5 siRNA significantly altered the abundance of p53 and p16 mRNA induced by DHA treatment (Figure 6d). Besides, the results from immunofluorescence assay showed that DHA as well as Atg5 plasmid treatment significantly increased the level of cellular GATA6 and p53 compared with untreated cells, whereas the treatment of cells with Atg5 siRNA, which downregulated the expression of cellular GATA6 and p53, dramatically diminished the effect of DHA or Atg5 plasmid in inducing cell senescence(Figure 6e). Additional experiments showed that DHA and Atg5 plasmid treatment significantly inhibited the telomerase activity, whereas the pretreatment with Atg5 siRNA abrogated DHA-induced inhibitory effects (Supplementary Figure 5A). Lastly, we examined the effect of Atg5 plasmid or Atg5 siRNA on cell cycle distribution. As shown in Supplementary Figures 5B–D, the pretreatment with Atg5 plasmid decreased the expression of Cyclin D1, CDK4 and CDK6, while siRNA-mediated knockdown of Atg5 dramatically upregulated their expression and resulted in a pronounced and significant attenuation of DHA-induced inhibitory effects. Collectively, these results support that autophagy activation mediates DHA-induced GATA6 accumulation and cell senescence in activated HSCs.

### Degradation of p62 is required for autophagy to mediate DHA-induced GATA6 accumulation and HSC senescence *in vitro*

**Figure 7:**
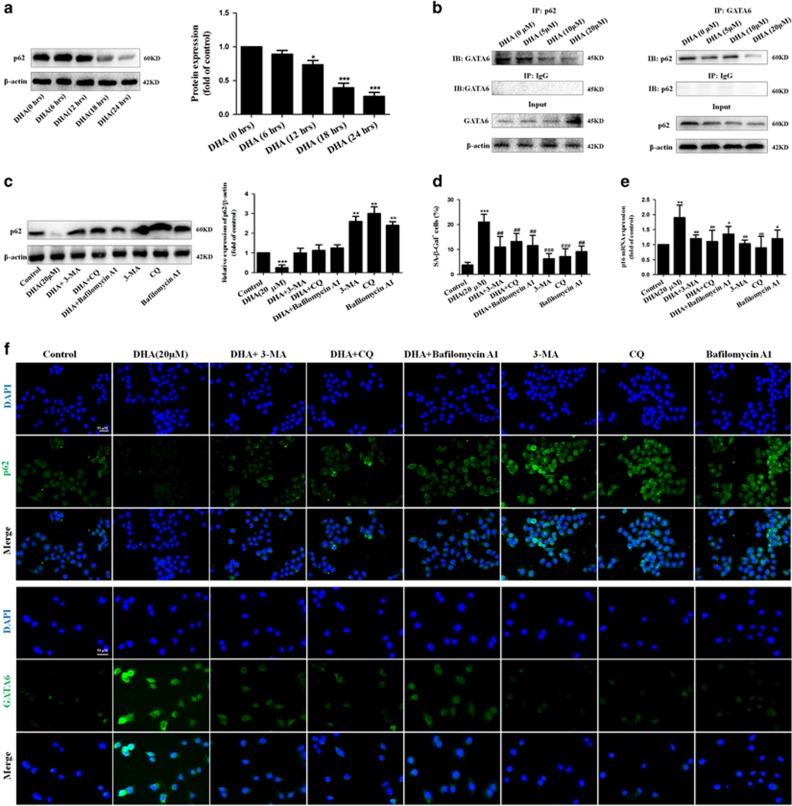
Degradation of p62 is required for autophagy to mediate DHA-induced GATA6 accumulation and HSC senescence *in vitro*. (**a**) Activated HSCs were treated with DHA at 20 *μ*M for various hours. Then, western blot was used to determine the expression of p62. (**b**) Immunoprecipitation assay was used to show the interaction of GATA6 and p62. (**c**) Activated HSCs were treated with 20 *μ*M DHA in the absence or presence of autophagy inhibitors 3-MA (10 mM), CQ (10 *μ*M), Bafilomycin A1 (5 nM). After 24 h incubation, the expression of p62 was determined by western blot analysis. (**d**) *β*-galactosidase staining analysis was used to detect HSC senescence. (**e**) Real-time PCR analyses were used to determine the expression of p16. (**f**) The expression of p62 and GATA6 was assessed by immunofluorescence. For the statistics of each panel in this figure, data are expressed as mean±S.D. (*n*=3); *P<0.05 *versus* control, **P<0.01 *versus* control, ***P<0.001 *versus* control. ^#^*P*<0.05 *versus* DHA treatment, ^##^*P*<0.01 *versus* DHA treatment, ^###^*P*<0.001 *versus* DHA treatment

To further investigate how DHA-induced autophagy promoted GATA6 accumulation, we hypothesized that the degradation of p62 had an important role in DHA-induced GATA6 accumulation and HSC senescence. To test this hypothesis, the status of this p62 protein was evaluated following the DHA treatment. Western blot analysis showed that treatment with DHA for 18 h resulted in a significant inhibitory effect, which was negatively correlated to the GATA6 accumulation of DHA-treated HSCs (Figure 7a). Then, the interaction between p62 and GATA6 was determined by immunoprecipitation assay. The result revealed that DHA blocked the binding of p62 to GATA6 in a dose-dependent manner (Figure 7b). Interestingly, these data suggest that p62 may be a negative regulator of GATA6 accumulation, but this regulation is suppressed by DHA-induced autophagy activation, thereby stabilizing GATA6. Next, various autophagy inhibitors, 3-MA, CQ and Bafilomycin A1, were used to induce p62 accumulation for a reverse verification. As shown in Figures 7c and f, treatment with DHA significantly decreased the expression of p62, whereas pretreatment with 3-MA, CQ, and Bafilomycin A1 completely abrogated DHA-induced p62 degradation. As expected, immunofluorescent staining of GATA6 demonstrated that the stabilization of p62 induced by autophagy inhibitors 3-MA, CQ and Bafilomycin A1, impaired DHA-induced GATA6 accumulation (Figure 7f). Furthermore, a panel of senescence-associated markers, including SA-*β*-gal, p53 and p21, were all determined. Unsurprisingly, treatment with 3-MA, CQ, and Bafilomycin A1 completely abrogated DHA-induced p62 degradation, and in turn, decreased the number of SA-*β*-Gal-positive HSCs (Figure 7d), and p16 mRNA expression (Figure 7e). More importantly, p62 overexpression plasmid also resulted in a pronounced and significant attenuation of DHA-induced GATA6 accumulation, and then, decreased the number of SA-*β*-Gal-positive HSCs, and p53 or p16 mRNA expression (Supplementary Figures 6A–D). Taken together, these data suggest that the degradation of p62 is required for autophagy to mediate DHA-induced GATA6 accumulation and HSC senescence *in vitro*.

## Discussion

Cellular senescence acts as a potent mechanism of tumor suppression.^17, 18, 19, 20^ However, its functional contribution to non-cancer pathologies has not been fully understood. Attractively, previous studies,^22, 40^ have discovered the existence of senescent HSCs during the development of liver fibrosis. Krizhanovsky *et al.* showed that senescent activated HSCs reduced the secretion of ECM components, enhanced immune surveillance, and facilitated the reversion of fibrosis.^40^ Kong *et al.* also reported that interleukin-22 induced HSC senescence and restricted liver fibrosis in mice.^41^ Consistent with previous studies,^40, 41^ we showed that the senescence of activated HSCs induced by DHA treatment provide a brake on the fibrogenic response to damage by limiting the expansion of the cell type responsible for producing the fibrotic scar. To our knowledge, this is the first report that DHA can induce HSC senescence to alleviate liver fibrosis.

Importantly, our study identified the transcription factor GATA6 as an upstream molecule in the facilitation of DHA-induced HSC senescence. The GATA family of transcription factors consists of six proteins (GATA1-6) which are involved in a variety of physiological and pathological processes.^42, 43^ Recently, Kang *et al.* reported that GATA4 functioned as a key switch in the senescence regulatory network to activate the senescence-associated secretory phenotype (SASP).^36^ In the present study, we found that the accumulation of GATA6 was required for DHA to induce HSC senescence *in vitro* and *in vivo*. siRNA-mediated knockdown of GATA6 dramatically abolished DHA-induced upregulation of p53 and p16, and in turn inhibited HSC senescence. Although our data suggested direct connection between GATA6 and DHA-induced HSC senescence, we could not eliminate other effects that may mediate the protective effect of DHA.

Autophagy and cellular senescence are stress responses essential for homeostasis.^24^ While recent studies indicate a genetic relationship between autophagy and senescence, whether autophagy acts positively or negatively on senescence is still subject to debate.^24, 25^ Garcia-Prat *et al.* reported that autophagy maintains stemness by preventing senescence.^44^ Conversely, Liu *et al.* revealed that autophagy suppresses melanoma tumorigenesis by inducing senescence.^45^ In the current study, we found that activation of autophagy is required for DHA to induce HSC senescence in live animal model and cell model. Down-regulation of autophagy activity, using Atg5 siRNA, led to an inhibition of DHA-induced HSC senescence, while Atg5 plasmid enhanced the effect of DHA *in vitro*. Attractively, we found that p62 may be a negative regulator of GATA6 accumulation, but this regulation was suppressed by DHA-induced autophagy activation. Treatment of cultured HSCs with various autophagy inhibitors or p62 overexpression plasmid, led to an inhibition of DHA-induced p62 degradation, and in turn, prevented DHA-induced GATA6 accumulation and HSC senescence. Although more experiments are needed to determine the exact role of autophagy in cell senescence, our results indicate a similar function in HSCs in consistent with previous reports.^24, 25^

**Figure 8:**
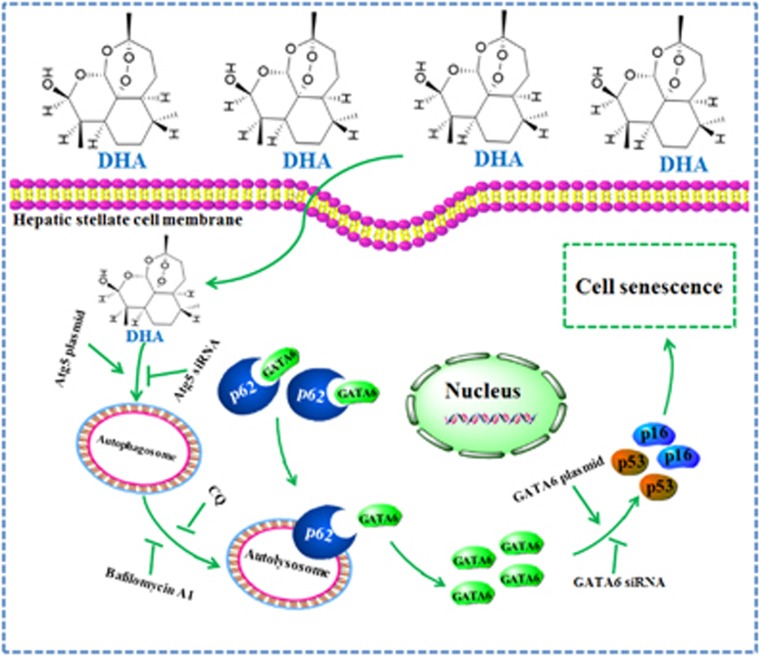
Schema of the underlying mechanism of DHA-induced HSC senescence. DHA treatment induces the accumulation of senescent activated HSCs in rat fibrotic liver, and promotes the expression of senescence markers p53, p16, p21 and Hmga1 in cell model. Importantly, GATA6 is identified as an upstream molecule to regulate p53 and p16 activation. siRNA-mediated knockdown of GATA6 dramatically abolishes DHA-induced activation of p53 and p16, and in turn inhibits HSC senescence. Interestingly, DHA also appears to increase the autophagosome generation and autophagic flux via an mTOR-dependent mechanism in activated HSCs, which are potential causes of DHA-induced GATA6 accumulation and HSC senescence. Autophagy depletion impairs GATA6 accumulation, while autophagy induction shows a synergistic effect with DHA. Attractively, p62 is found to act as a negative regulator of GATA6 accumulation, but this regulation is suppressed by DHA treatment, thereby stabilizing GATA6.Treatment of cultured HSCs with the autophagy inhibitors, led to an inhibition of DHA-induced p62 degradation, and in turn, prevents DHA-induced GATA6 accumulation and HSC senescence

Overall, these results provide the first mechanistic evidence that interaction between autophagy and senescence is required for DHA to alleviate liver fibrosis (Figure 8). Since there still are no clinically effective anti-fibrosis drugs, understanding the mechanistic basis of action of natural dietary products such as DHA offers further insight into developing drugs for the prevention and treatment of liver fibrosis.

## Materials and methods

### Reagents and antibodies

DHA, colchicine, PDGF-BB, Etoposide, CQ, rapamycin (Rapa), 3-MA, bafilomycin A1, dimethyl sulfoxide (DMSO), anti-rabbit IgG, and anti-mouse IgG were purchased from Sigma-Aldrich (St. Louis, MO, USA). Dulbecco’s modified essential medium (DMEM), Opti MEM medium, phosphate-buffered saline (PBS), trypsin-EDTA and fetal bovine serum (FBS) were bought from GIBCO BRL (Grand Island, NY, USA). Primary antibodies against p53, p16, p21, *α*-SMA, Hmga1, LC3-I/II, ULK1, p-ULK1, mTOR, p-mTOR, Atg3, Atg5-Atg12, Atg6, Atg7, Atg14, p62, *β*-galactosidase and *β*-actin were purchased from Cell Signaling Technology (Danvers, MA, USA). Primary antibody against *α*1(I) procollagen was purchased from Epitomics (San Francisco, CA, USA). Primary antibodies against Ki67, p62 and GATA6 were purchased from Abcam Technology (Abcam, Cambridge, UK). Atg5 siRNA, GATA6 siRNA, negative control siRNA, Atg5 plasmid, GATA6 plasmid, negative control vectors and mRFP-GFP-LC3 plasmid were purchased from Hanbio (Shanghai, China). MegaTran 1.0 transfection reagent was from OriGene (Rockville, MD, USA).

### Animals and experimental design

All experimental procedures were approved by the institutional and local committee on the care and use of animals of Nanjing University of Chinese Medicine (Nanjing, China), and all animals received humane care according to the National Institutes of Health (USA) guidelines. Male Sprague-Dawley rats weighing approximately 180–220 g were procured from Nanjing Medical University (Nanjing, China). A mixture of CCl_4_ (0.1 ml/100g bodyweight) and olive oil (1:1 (w/v)) was used to induce liver fibrosis in rats. Fifty rats were randomly divided into five groups of ten animals each with comparable mean bodyweight. Rats of Group 1 were served as a vehicle control and intraperitoneally (i.p.) injected with olive oil. Rats of group 2 were i.p. injected with CCl_4_. Rats of Groups 3, 4 and 5 were served as treatment groups and i.p. injected by CCl_4_ and DHA with 3.5, 7 and 14 mg/kg, respectively. Rats of groups 2–5 were i.p. injected with CCl_4_ every other day for 8 weeks. DHA was suspended in sterile PBS and given once daily by intraperitoneal injection during weeks 5–8. At the end of the experiment, rats were sacrificed after anesthetization with an injection of 50 mg/kg pentobarbital. A small portion of the liver was removed for histopathological and immunohistochemical studies.

Male ICR mice (ages 6–8 weeks) were purchased from Nanjing Medical University (Nanjing, China). Seventy mice were randomly divided into seven groups of ten animals each with comparable mean bodyweight. Mice of seven groups were administrated with Vehicle control, CCl_4_, CCl_4_+Ad.Fc (a control adenovirus encoding IgG2 *α* Fc fragment), CCl_4_+DHA (20 mg/kg, once a day), CCl_4_+Ad.Fc+DHA, CCl_4_+Ad.shGATA6 (adenovirus encoding mouse GATA6 shRNA for inhibiting GATA6 expression) or CCl_4_+Ad.shGATA6+DHA, respectively, throughout the 8-week period of CCl_4_ treatment. Adenoviruses (2.5 × 10^7^pfu/g, once per 2 weeks) were injected into mice by tail vein. A mixture of carbon tetrachloride (CCl_4_; 0.5 ml per 100 g bodyweight) and olive oil (1: 9 (v/v)) was used to induce liver fibrosis in mice by i.p. injection. After 8 weeks, liver were fixed in 4% buffered paraformaldehyde for histological analysis of liver fibrosis and immunostaining analysis or HSCs were isolated for western blot analysis.

### Histological analysis

Hematoxylin and eosin, Sirius Red and Masson staining were performed on 4-*μ*m thick formalin-fixed paraffin-embedded tissue sections. Sirius Red and Masson-stained areas from 10 fields (magnification × 200) from 3 to 6 mice/group were quantified with Image J.

### Cell isolation, cell culture conditions and drug treatment

Primary rat HSCs were isolated from male Sprague-Dawley rats weighing approximately 180–220 g (Nanjing Medical University, Nanjing, China) as described.^27^ Isolated HSCs were cultured in DMEM with 10% fetal bovine serum, 1% antibiotics and maintained at 37 °C in a humidified incubator of 5% CO_2_ and 95% air. Cell morphology was assessed using an inverted microscope with a Leica Qwin System (Leica, Germany). DHA was dissolved in DMSO at a concentration of 10 mM and was stored in a dark colored bottle at −20 °C. The stock was diluted to required concentration with DMSO when needed. Before the DHA treatment, cells were grown to about 70% confluence, and then exposed to DHA at different concentrations (0–20 *μ*M) for different period of time (0–24 h). Cells grown in a medium containing an equivalent amount of DMSO without DHA were served as control.

### Plasmid transfection

Atg5 siRNA, GATA6 siRNA, p62 siRNA, negative control siRNA, Atg5 plasmid, GATA6 plasmid, p62 plasmid, negative control vectors and mRFP-GFP-LC3 plasmid were transfected into HSCs using MegaTran 1.0 transfection reagent according to manufacturer's instructions.^12^ After 24 h, cells were treated with selenite or PBS as a solution control. The transfection efficiency was confirmed by western blot analysis.

### RNA isolation and real-time PCR

Total RNA was isolated and qPCR performed using the QuantiTect SYBR Green PCR Kit (Qiagen, Valencia, CA, USA) in accordance to the manufacturer's instructions.^12^ Actin levels were taken for normalization and fold change was calculated using 2^-ddCt^. Primer Sequence available on request.

### Western blot analysis

Cells or tissue samples were lysed using mammalian lysis buffer (Sigma, St. Louis, MO, USA) and immunoblotting was performed as per the manufacturer’s guidelines^12^ (Bio/Rad, Hercules, CA, USA). Briefly, the protein levels were determined using a BCA assay kit (Pierce, Rockford, IL, USA). Proteins (50 *μ*g/well) were separated by SDS-polyacrylamide gel, transferred to a PVDF membrane (Millipore, Burlington, MA, USA), blocked with 5% skim milk in Tris-buffered saline containing 0.1% Tween 20. Target proteins were detected by corresponding primary antibodies, and subsequently by horseradish peroxidase-conjugated secondary antibodies. Protein bands were visualized using chemiluminescence reagent (Millipore). Equivalent loading was confirmed using an antibody against *β*-actin. Densitometry analysis was performed using Image J software (NIH, Bethesda, MD, USA).

### Immunoprecipitation assay

An immunoprecipitation assay was performed using extracts of the activated HSCs as previously described.^46^ Briefly, immunoprecipitation was performed using Classic Magnetic IP/Co-IP Kit (Pierce, Carlsbad, CA, USA) to analyze the interaction between GATA6 and p62 (Abcam, Cambridge, UK). Activated HSCs were washed 3 times in PBS and lysed in IP Lysis Buffer (Abcam) on ice for 5 min. The protein lysate was collected by centrifugation. Protein A/G Magnetic Beads (25 *μ*l) were incubated with anti-GATA6 antibody (Abcam) for 1 h at room temperature, and then added to the protein lysate and incubated overnight at 4 °C. The beads were then collected and washed in IP Wash Buffer for 5 times. Proteins were dissolved in Elution Buffer and detected by western blot.

### Long-lived proteins degradation analysis

Primary HSCs were cultured in DMEM (GIBCO BRL) supplemented with 100 units/ml penicillin, 100 *μ*g/ml streptomycin, glutamine, 10% fetal bovine serum (GIBCO BRL) and labeled with either l-arginine and l-lysine, l-[U-^13^C_6_,^14^N_4_] arginine and l-[^2^H_4_]lysine, or l-[U-^13^C_6_,^15^N_4_]arginine and l-[U-^13^C_6_,^15^N_2_]lysine (Cambridge Isotope Laboratories, Andover, MA, USA; Sigma-Aldrich) (2*15-cm cell culture dishes per condition; ~95% confluent). Cells were washed three times in PBS before they were treated with DMSO or 20 *μ*M DHA following 20 ng/ml PDGF-BB treatment for indicated hours. All treatments were carried out at 37 °C. After drug treatment, the cells were scraped in cell scraping buffer (0.25 M sucrose, 1 mM sodium ortho-vanadate, 5 mM NaF, 5 mM *β*-glycerophosphate, and protease inhibitor mixture (Complete TM tablets, Roche Diagnostics)) and normalized by cell counting. Mixed cells were centrifuged for 5 min at 1800 rpm and lysed in 6 M urea and 2 M thiourea, and 2% Benzonase (Merck) was added before samples were concentrated on spin tubes (cutoff, 500 Da). Protein mixtures were separated by SDS-PAGE (4–12% bis-Tris gra-dient gel, NuPAGE, Invitrogen). Gel lanes were cut into 15 slices, and samples were in-gel digested, and resulting peptide mixtures were STAGE-tipped. Relative quantification and identification of peptides were analyzed by LC–MS/MS as described previously.^39^

### Transmission electron microscopy

Cells were seeded onto 4-chambered coverglass (Lab-Tek Chambered Coverglass System) (Nalgene/Nunc, Rochester, NY, USA) at a density of 2 × 10^4^ cells/ml (14 000 cells/well). Images were acquired using the Olympus EM208S transmission electron microscope.

### Immunofluorescence analysis

Immunofluorescence staining with liver tissues or treated cells were performed as we previously reported.^12^ 4′,6-Diamidino-2-phenylindole (DAPI) was used to stain the nucleus in liver tissues and HSCs. All the images were captured with the fluorescence microscope and representative images were shown. The software Image J was used to quantitate the fluorescent intensity on the micrographs.

### Analysis of HSC senescence

HSC senescence was determined by the detection of SA-*β*-gal (senescence-associated *β*-galactosidase) activity using an SA-*β*-gal staining kit (Cell Signaling). Briefly, adherent cells were fixed with 0.5% glutaraldehyde in PBS for 15 min, washed with PBS containing 1 mM MgCl_2_ and stained overnight in PBS containing 1 mM MgCl_2_, 1 mg/ml X-Gal, 5 mM potassium ferricyanide and 5 mM potassium ferrocyanide. All the images were captured with a light microscope and representative images were shown. Results were from triplicate experiments.

### Cell cycle analysis by flow cytometry

Distribution of cell cycle was determined by PI staining and flow cytometry analysis. HSCs were seeded in six-well plates and cultured in DMEM supplemented with 10% FBS for 24 h, and then were treated with DMSO, Etoposide and DHA at indicated concentrations for 24 h. Cells were then harvested and fixed, and the cell cycle was then detected by the cellular DNA flow cytometric analysis kit (Nanjing Keygen Biotech) according to the protocol.^22^ Percentages of cells within cell cycle compartments (G0/G1, S and G2/M) were determined by flow cytometry (FACS Calibur; Becton, Dickinson and Company, Franklin Lakes, NJ, USA). The data were analyzed using the software Cell Quest. Results were from triplicate experiments.

### Calculations and statistics

Individual culture experiments and animal experiments were performed in duplicate or triplicate and repeated three times using matched controls, and the data were pooled. Results were expressed as either S.D. or mean±standard error of the mean (S.E.M.). The statistical significance of differences (*P<0.05) was assessed by *t*-test.

## References

[bib1] Lee YA, Wallace MC, Friedman SL. Pathobiology of liver fibrosis: a translational success story. Gut 2015; 64: 830–841. 25681399 10.1136/gutjnl-2014-306842 PMC4477794

[bib2] Kang N, Gores GJ, Shah VH. Hepatic stellate cells: partners in crime for liver metastases? Hepatology 2011; 54: 707–713. 21520207 10.1002/hep.24384 PMC3145026

[bib3] Kitano M, Bloomston PM. Hepatic stellate cells and microRNAs in pathogenesis of liver fibrosis. J Clin Med 2016; 5: 10–13. 10.3390/jcm5030038 PMC4810109 26999230

[bib4] Lambrecht J, Mannaerts I, van Grunsven LA. The role of miRNAs in stress-responsive hepatic stellate cells during liver fibrosis. Front Physiol 2015; 6: 209. 26283969 10.3389/fphys.2015.00209 PMC4516870

[bib5] Thompson AI, Conroy KP, Henderson NC. Hepatic stellate cells: central modulators of hepatic carcinogenesis. BMC Gastroenterol 2015; 15: 63. 26013123 10.1186/s12876-015-0291-5 PMC4445994

[bib6] Carloni V, Luong TV, Rombouts K. Hepatic stellate cells and extracellular matrix in hepatocellular carcinoma: more complicated than ever. Liver Int 2014; 34: 834–843. 24397349 10.1111/liv.12465

[bib7] Zhang Z, Guo Y, Zhang S, Zhang Y, Wang Y, Ni W et al. Curcumin modulates cannabinoid receptors in liver fibrosis *in vivo* and inhibits extracellular matrix expression in hepatic stellate cells by suppressing cannabinoid receptor type-1 *in vitro*. Eur J Pharmacol 2013; 721: 133–140. 24076327 10.1016/j.ejphar.2013.09.042

[bib8] Zhang F, Zhang Z, Kong D, Zhang X, Chen L, Zhu X et al. Tetramethylpyrazine reduces glucose and insulin-induced activation of hepatic stellate cells by inhibiting insulin receptor-mediated PI3K/AKT and ERK pathways. Mol Cell Endocrinol 2014; 382: 197–204. 24071517 10.1016/j.mce.2013.09.020

[bib9] Tai X, Cai XB, Zhang Z, Wei R. *In vitro* and *in vivo* inhibition of tumor cell viability by combined dihydroartemisinin and doxorubicin treatment, and the underlying mechanism. Oncol Lett 2016; 12: 3701–3706. 27900057 10.3892/ol.2016.5187 PMC5104152

[bib10] Wu C, Liu J, Pan X, Xian W, Li B, Peng W et al. Design, synthesis and evaluation of the antibacterial enhancement activities of amino dihydroartemisinin derivatives. Molecules 2013; 18: 6866–6882. 23752470 10.3390/molecules18066866 PMC6270293

[bib11] Zhang XG, Li GX, Zhao SS, Xu FL, Wang YH, Wang W. A review of dihydroartemisinin as another gift from traditional Chinese medicine not only for malaria control but also for schistosomiasis control. Parasitol Res 2014; 113: 1769–1773. 24609234 10.1007/s00436-014-3822-z

[bib12] Zhang Z, Guo M, Zhao S, Shao J, Zheng S. ROS-JNK1/2-dependent activation of autophagy is required for the induction of anti-inflammatory effect of dihydroartemisinin in liver fibrosis. Free Radic Biol Med 2016; 101: 272–283. 27989749 10.1016/j.freeradbiomed.2016.10.498

[bib13] Chen Q, Chen L, Kong D, Shao J, Wu L, Zheng S. Dihydroartemisinin alleviates bile duct ligation-induced liver fibrosis and hepatic stellate cell activation by interfering with the PDGF-betaR/ERK signaling pathway. Int Immunopharmacol 2016; 34: 250–258. 27038258 10.1016/j.intimp.2016.03.011

[bib14] Chen Q, Chen L, Wu X, Zhang F, Jin H, Lu C et al. Dihydroartemisinin prevents liver fibrosis in bile duct ligated rats by inducing hepatic stellate cell apoptosis through modulating the PI3K/Akt pathway. IUBMB Life 2016; 68: 220–231. 26865509 10.1002/iub.1478

[bib15] Xu W, Lu C, Zhang F, Shao J, Zheng S. Dihydroartemisinin restricts hepatic stellate cell contraction via an FXR-S1PR2-dependent mechanism. IUBMB Life 2016; 68: 376–387. 27027402 10.1002/iub.1492

[bib16] Xu W, Lu C, Yao L, Zhang F, Shao J, Zheng S. Dihydroartemisinin protects against alcoholic liver injury through alleviating hepatocyte steatosis in a farnesoid X receptor-dependentmanner. Toxicol Appl Pharmacol 2016; 315: 23–34. 27939985 10.1016/j.taap.2016.12.001

[bib17] Kaul Z, Cesare AJ, Huschtscha LI, Neumann AA, Reddel RR. Five dysfunctional telomeres predict onset of senescence in human cells. EMBO Rep 2012; 13: 52–59. 10.1038/embor.2011.227 PMC3246253 22157895

[bib18] Shao AW, Sun H, Geng Y, Peng Q, Wang P, Chen J et al. Bclaf1 is an important NF-κB signaling transducer and C/EBPbeta regulator in DNA damage-induced senescence. Cell Death Differ 2016; 23: 865–875. 26794446 10.1038/cdd.2015.150 PMC4832105

[bib19] Kagawa S, Natsuizaka M, Whelan KA, Facompre N, Naganuma S, Ohashi S et al. Cellular senescence checkpoint function determines differential Notch1-dependent oncogenic and tumor-suppressor activities. Oncogene 2016; 34: 2347–2359. 10.1038/onc.2014.169 PMC4268095 24931169

[bib20] Vicente R, Mausset-Bonnefont AL, Jorgensen C, Louis-Plence P, Brondello JM. Cellular senescence impact on immune cell fate and function. Aging Cell 2016; 15: 400–406. 26910559 10.1111/acel.12455 PMC4854915

[bib21] Rufini A, Tucci P, Celardo I, Melino G. Senescence and aging: the critical roles of p53. Oncogene 2013; 32: 5129–5143. 23416979 10.1038/onc.2012.640

[bib22] Jin H, Lian N, Zhang F, Chen L, Chen Q, Lu C et al. Activation of PPARγ/P53 signaling is required for curcumin to induce hepatic stellate cell senescence. Cell Death Dis 2016; 7: e2189. 27077805 10.1038/cddis.2016.92 PMC4855671

[bib23] Romagosa C, Simonetti S, Lopez-Vicente L, Mazo A, Lleonart ME, Castellvi J et al. p16(Ink4a) overexpression in cancer: a tumor suppressor gene associated with senescence and high-grade tumors. Oncogene 2011; 30: 2087–2097. 21297668 10.1038/onc.2010.614

[bib24] Zhang H, Puleston DJ, Simon AK. Autophagy and immune senescence. Trends Mol Med 2016; 22: 671–686. 27395769 10.1016/j.molmed.2016.06.001

[bib25] Kang C, Elledge SJ. How autophagy both activates and inhibits cellular senescence. Autophagy 2016; 12: 898–899. 27129029 10.1080/15548627.2015.1121361 PMC4854549

[bib26] Lechuga CG, Hernández-Nazara ZH, Hernández E, Bustamante M, Desierto G, Cotty A et al. PI3K is involved in PDGF-beta receptor upregulation post-PDGF-BB treatment in mouse HSC. Am J Physiol Gastrointest Liver Physiol 2006; 291: G1051–G1061. 16990448 10.1152/ajpgi.00058.2005

[bib27] Bartneck M, Warzecha KT, Tag CG, Sauer-Lehnen S, Heymann F, Trautwein C et al. Isolation and time lapse microscopy of highly pure hepatic stellate cells. Anal Cell Pathol 2015; 2015: 417023. 10.1155/2015/417023 PMC4519541 26258009

[bib28] Kim KM, Kim JM, Yoo YH, Kim JI, Park YC. Cilostazol induces cellular senescence and confers resistance to etoposide-induced apoptosis in articular chondrocytes. Int J Mol Med 2012; 29: 619–624. 22294024 10.3892/ijmm.2012.892 PMC3577138

[bib29] Truman AW, Kristjansdottir K, Wolfgeher D, Hasin N, Polier S, Zhang H et al. CDK-dependent Hsp70 Phosphorylation controls G1 cyclin abundance and cell-cycle progression. Cell 2012; 151: 1308–1318. 23217712 10.1016/j.cell.2012.10.051 PMC3778871

[bib30] Cassidy LD, Narita M. GATA get a hold on senescence. Science 2015; 349: 1448–1449. 26404812 10.1126/science.aad2501

[bib31] El Hasasna H, Athamneh K, Al Samri H, Karuvantevida N, Al Dhaheri Y, Hisaindee S et al. Rhus coriaria induces senescence and autophagic cell death in breast cancer cells through a mechanism involving p38 and ERK1/2 activation. Sci Rep 2015; 5: 13013. 26263881 10.1038/srep13013 PMC4532997

[bib32] Kim KH, Park B, Rhee DK, Pyo S. Acrylamide induces senescence in macrophages through a process involving ATF3, ROS, p38/JNK, and a telomerase-independent pathway. Chem Res Toxicol 2015; 28: 71–86. 25531190 10.1021/tx500341z

[bib33] Kim JE, Jin DH, Lee SD, Hong SW, Shin JS, Lee SK et al. Vitamin C inhibits p53-induced replicative senescence through suppression of ROS production and p38 MAPK activity. Int J Mol Med 2008; 22: 651–655. 18949386

[bib34] Fan T, Huang Z, Chen L, Wang W, Zhang B, Xu Y et al. Associations between autophagy, the ubiquitin-proteasome system and endoplasmic reticulum stress in hypoxia-deoxygenation or ischemia-reperfusion. Eur J Pharmacol 2016; 791: 157–167. 27568838 10.1016/j.ejphar.2016.08.026

[bib35] Levchenko M, Lorenzi I, Dudek J. The degradation pathway of the mitophagy receptor Atg32 is re-routed by a posttranslational modification. PLoS One 2016; 11: e0168518. 27992522 10.1371/journal.pone.0168518 PMC5161373

[bib36] Kang C, Xu Q, Martin TD, Li MZ, Demaria M, Aron L et al. The DNA damage response induces inflammation and senescence by inhibiting autophagy of GATA4. Science 2015; 349: aaa5612. 26404840 10.1126/science.aaa5612 PMC4942138

[bib37] Tang BL. mTOR, autophagy, and reprogramming. Front Cell Dev Biol 2014; 1: 4. 25364709 10.3389/fcell.2013.00004 PMC4207015

[bib38] Maiese K. Targeting molecules to medicine with mTOR, autophagy and neurodegenerative disorders. Br J Clin Pharmacol 2016; 82: 1245–1266. 26469771 10.1111/bcp.12804 PMC5061806

[bib39] Klionsky DJ, Abdelmohsen K, Abe A, Abedin MJ, Abeliovich H, Acevedo Arozena A et al. Guidelines for the use and interpretation of assays for monitoring autophagy (3rd edition). Autophagy 2016; 12: 1–222. 26799652 10.1080/15548627.2015.1100356 PMC4835977

[bib40] Krizhanovsky V, Yon M, Dickins RA, Hearn S, Simon J, Miething C et al. Senescence of activated stellate cells limits liver fibrosis. Cell 2008; 134: 657–667. 18724938 10.1016/j.cell.2008.06.049 PMC3073300

[bib41] Kong X, Feng D, Wang H, Hong F, Bertola A, Wang FS et al. Interleukin-22 induces hepatic stellate cell senescence and restricts liver fibrosis in mice. Hepatology 2012; 56: 1150–1159. 22473749 10.1002/hep.25744 PMC3394879

[bib42] Lentjes MH, Niessen HE, Akiyama Y, de Bruïne AP, Melotte V, van Engeland M. The emerging role of GATA transcription factors in development and disease. Expert Rev Mol Med 2016; 18: e3. 26953528 10.1017/erm.2016.2 PMC4836206

[bib43] Block DH, Shapira M. GATA transcription factors as tissue-specific master regulators for induced responses. Worm 2015; 4: e1118607. 27123374 10.1080/21624054.2015.1118607 PMC4826149

[bib44] García-Prat L, Martínez-Vicente M, Perdiguero E, Ortet L, Rodríguez-Ubreva J, Rebollo E et al. Autophagy maintains stemness by preventing senescence. Nature 2016; 529: 37–42. 26738589 10.1038/nature16187

[bib45] Liu H, He Z, Simon HU. Autophagy suppresses melanoma tumorigenesis by inducing senescence. Autophagy 2014; 10: 372–373. 24300435 10.4161/auto.27163 PMC5396100

[bib46] Zhang Z, Zhao S, Yao Z, Wang L, Shao J, Chen A et al. Autophagy regulates turnover of lipid droplets via ROS-dependent Rab25 activation in hepatic stellate cell. Redox Biol 2017; 11: 322–334. 28038427 10.1016/j.redox.2016.12.021 PMC5199192

