# Supplementary material for: Interaction between autophagy and senescence is required for dihydroartemisinin to alleviate liver fibrosis
Source: Cell Death Dis. 2017 Jun 15;8(6):e2886–. doi: 10.1038/cddis.2017.255 (PMC5520911; doi:10.1038/cddis.2017.255)
Supplement: Supplementary Figure Legends [file cddis2017255x1.docx]

**Supplementary Figure legends**

**Supplementary Figure 1. DHA protects the liver against CCl_4_-induced injury and suppressed hepatic fibrogenesis in the rat model.** Rats were grouped as follows: group 1, vehicle control (no CCl_4_, no treatment); group 2, model group (with CCl_4_, no treatment); group 3, DHA (3.5 mg/kg) and CCl_4_-treated group; group 4, DHA (7 mg/kg) and CCl_4_-treated group; group 5, DHA (14mg/kg) and CCl_4_-treated group; group 6, colchicine and CCl_4_-treated group. **(A)** Liver sections were stained with hematoxylin and eosin, Masson reagents and Sirius red. Representative photographs are shown. **(B)** Determination of serum ALT, AST, and ALP levels was used to detect liver injury. **(C)** Liver/body weight ratio was detected to show the degree of liver injury induced CCl_4_. **(D)** β-galactosidase staining analysis were used to determine HSC senescence. For the statistics of each panel in this figure, data are expressed as mean±SD (n=6); ^##^P < 0.01 versus control, ^###^P < 0.001 versus control. *P < 0.05 versus the model group, **P < 0.01 versus the model group, ***P< 0.001 versus the model group.

**Supplementary Figure 2. DHA promotes activated HSC senescence in vitro.** Primary HSCs were exposed for 6 hours to 20ng/ml PDGF-BB followed by vehicle, Etoposide or DHA treatment at indicated concentrations for 24 h. **(A)** Light microscope photographing morphological changes of HSCs. **(B, C)** Real-time PCR and Western blot data showing levels of α-SMA (acta-2), Fibronectin, Procollagen 1α1 (procol1α1), TNF-α, and TGF-β **(D)** Real-time PCR analysis showing the mRNA levels of telomerase (TERT). **(E, F)** Cell cycle progression was measured using flow cytometry. **(G)** Real-time PCR analyses of cell cycle-regulatory proteins cyclin D1, cyclin E1, and CDK4. For the statistics of each panel in this figure, data are expressed as mean±SD (n=3); ^#^P < 0.05 versus control, ^##^P < 0.01 versus control, ^###^P < 0.001 versus control. *P < 0.05 versus the PDGF-BB treated HSCs, **P < 0.01 versus the PDGF-BB treated HSCs, ***P< 0.001 versus the PDGF-BB treated HSCs.

**Supplementary Figure 3. The accumulation of GATA6 is required for DHA to induce HSC senescence in vitro.** **(A-D)** Activated HSCs were stably transfected withGATA6 siRNA or GATA6 plasmid, and then were treated with the indicated concentration of DHA for 24 h. P53 and p16 immunostaining were used to determine the expression of p53 and p16 in HSCs treated with indicated reagents. **(E)** The expression level of phospho-ERK1/2, ERK1/2, phospho-JNK1/2, JNK1/2, phospho-p38, and p38 were detected by Western blot analysis. **(F)** Cells were pretreated with SP600125 (20 µM) for 1 h following by Control Vector or GATA6 plasmid treatment. Western blot analysis of p16 and p53 were performed. *P<0.05 versus GATA6 plasmid, **P<0.01 versus GATA6 plasmid, ***P<0.001 versus GATA6 plasmid **(G)** Cells were pretreated with SP600125 (20 µM) for 1 h followed by DHA treatment for 24 h. Following the treatment, Real-time PCR was used to determine the mRNA expression of p53, p16, and p21. *P < 0.05 versus control, **P < 0.01 versus control, ***P< 0.001 versus control. ^#^P < 0.05 versus DHA treatment, ^##^P < 0.01 versus DHA treatment, ^###^P < 0.001 versus DHA treatment

**Supplementary Figure 4. DHA increases the autophagosome generation and autophagic flux in activated HSCs. (A)** Activated HSCs were treated with DHA at various concentrations for 24 hr or DHA at 20μM for various hours. Then, Western blot and densitometric analysis were used to show the expression of p-mTOR, mTOR, p-ULK1, and ULK1. **(B)** Western blot and densitometric analysis were used to determine the effects of CQ (10μM) or DHA (20μM) on LC3-II in primary HSCs exposed PDGF-BB. **(C, D)** LC3-II and Atg6 immunostaining was used to show the increased endogenous LC3-II and Atg6 in HSCs exposed to 20 ng/mL PDGF-BB and DHA (20μM) for 24 h compared to vs. vehicles treated cells (Control). For the statistics of each panel in this figure, data are expressed as mean±SD (n=3); *P < 0.05 versus control, **P < 0.01 versus control, ***P< 0.001 versus control. ^##^P < 0.01 versus DHA treatment.

**Supplementary Figure 5. Disruption of autophagy impairs DHA-induced GATA6 accumulation and HSC senescence in vitro.** Activated HSCs were stably transfected with Atg5 siRNA or Atg5 plasmid, and then were treated with the indicated concentration of DHA for 24 h. **(A)** Real-time PCR analysis was used to determine the mRNA levels of telomerase (TERT). **(B-D)** Real-time PCR analysis was used to determine the mRNA levels of cyclin D1, CDK4, and CDK6. For the statistics of each panel in this figure, data are expressed as mean±SD (n=3); *P < 0.05 versus control, **P < 0.01 versus control, ***P< 0.001 versus control.

**Supplementary Figure 6. Degradation of p62 is required for autophagy to mediate DHA-induced GATA6 accumulation and HSC senescence in vitro.** Activated HSCs were stably transfected with p62 siRNA or p62 plasmid, and then were treated with the indicated concentration of DHA for 24 h. **(A, B)** Western blot was used to determine the expression of p62. **(C, D)** Real-time PCR was used to show the mRNA expression of GATA6, p53, and p16. β-galactosidase staining analysis were used to determine HSC senescence. For the statistics of each panel in this figure, data are expressed as mean±SD (n=3); *P < 0.05 versus control, **P < 0.01 versus control, ***P< 0.001 versus control. ^#^P < 0.05 versus DHA treatment, ^##^P < 0.01 versus DHA treatment, ^###^P < 0.001 versus DHA treatment
