# Supplementary figures and images for: Interaction between autophagy and senescence is required for dihydroartemisinin to alleviate liver fibrosis
Source: Cell Death Dis. 2017 Jun 15;8(6):e2886–. doi: 10.1038/cddis.2017.255 (PMC5520911; doi:10.1038/cddis.2017.255)

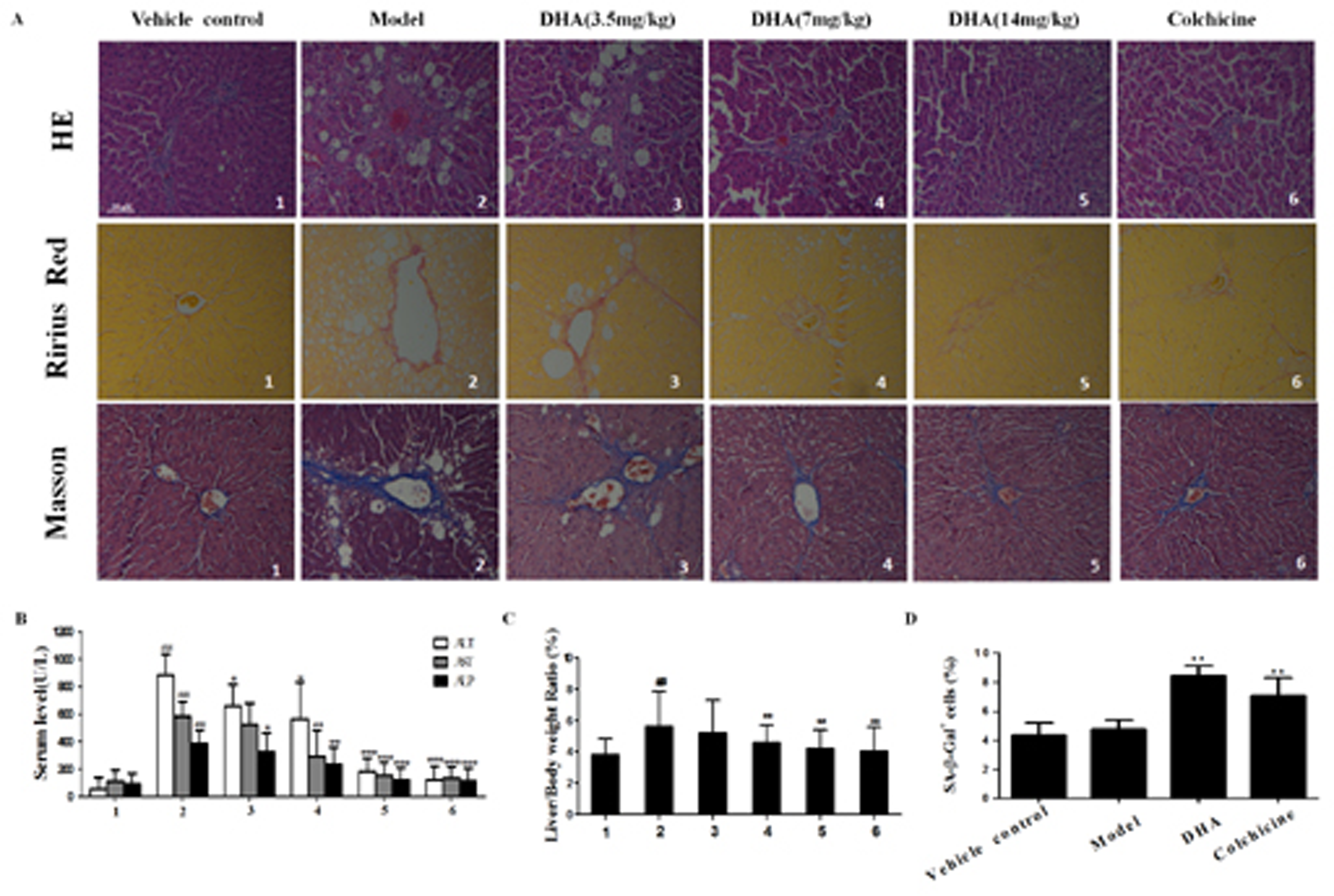

Supplement: Supplementary Figure S1 [file cddis2017255x2.tif]

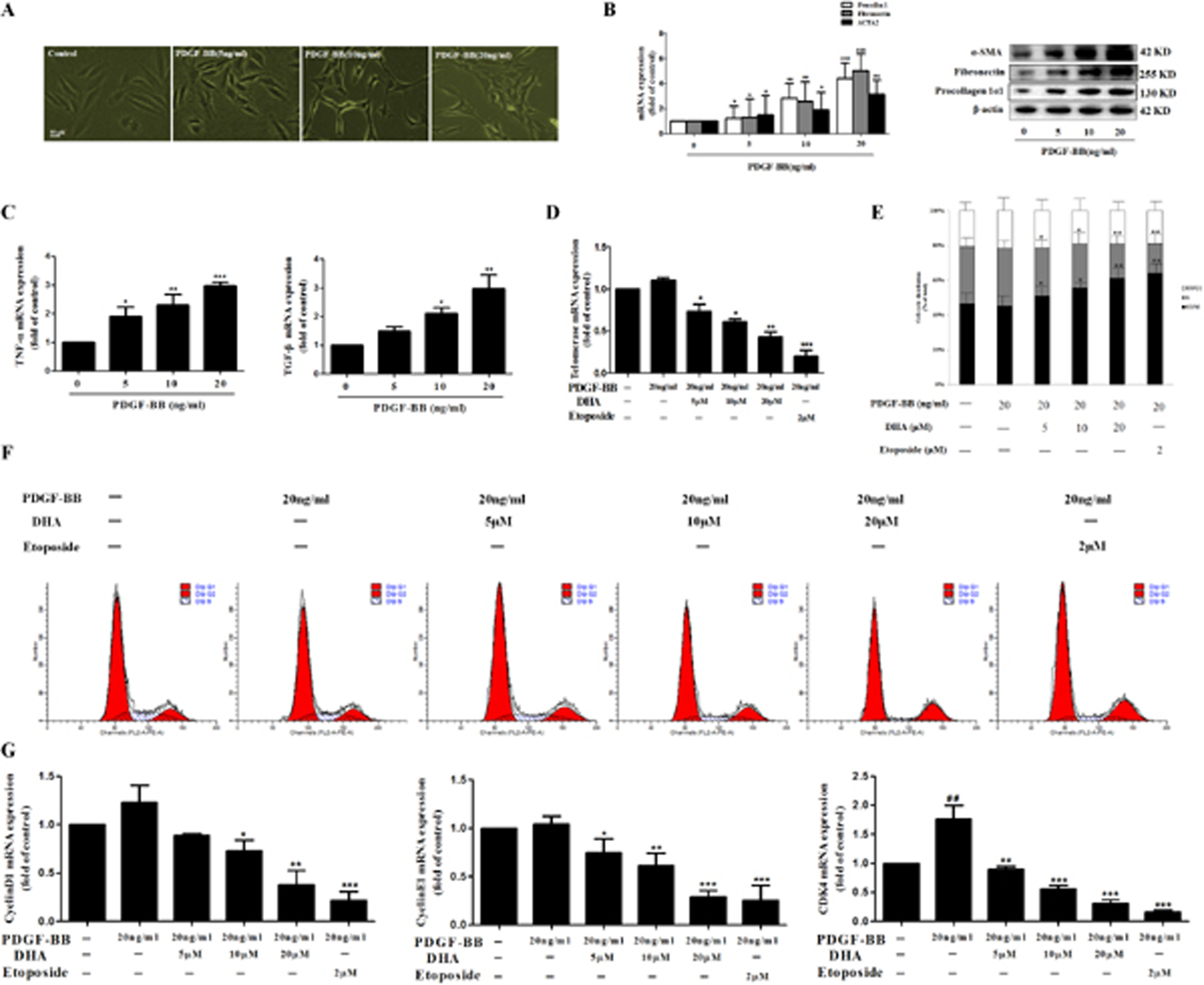

Supplement: Supplementary Figure S2 [file cddis2017255x3.tif]

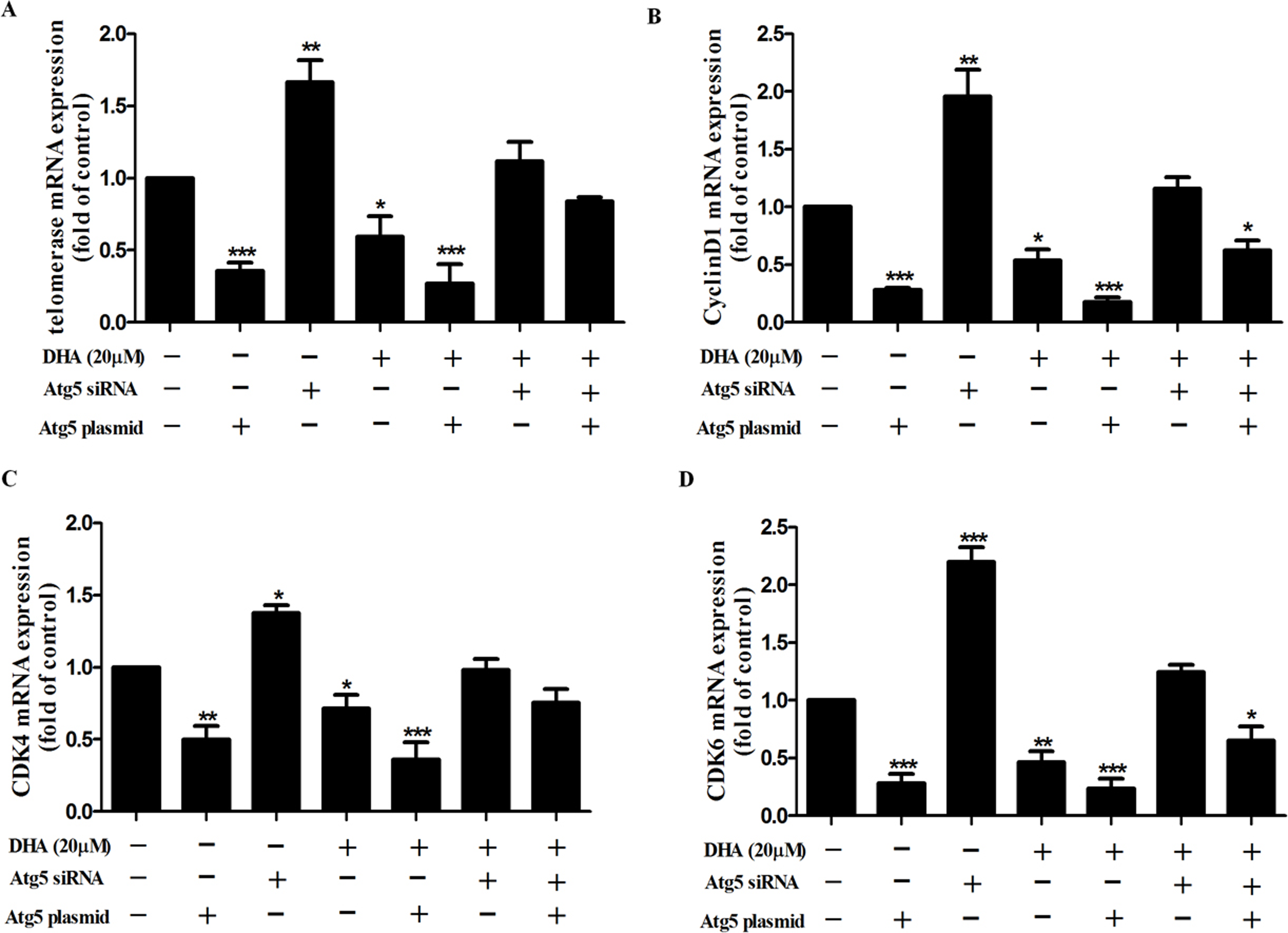

Supplement: Supplementary Figure S5 [file cddis2017255x6.tif]
